# Design and implementation of a natural language processing system at the point of care: MiADE (medical information AI data extractor)

**DOI:** 10.1186/s12911-025-03195-1

**Published:** 2025-10-07

**Authors:** Jennifer Jiang-Kells, James Brandreth, Leilei Zhu, Jack Ross, Yogini Jani, Enrico Costanza, Maisarah Amran, Zeljko Kraljevic, Xi Bai, M.M.N.S. Dilan, Jayathri Wijayarathne, Ravi Wickramaratne, Folkert W. Asselbergs, Richard J.B. Dobson, Wai Keong Wong, Anoop D. Shah

**Affiliations:** 1 Institute of Health Informatics, University College London, 222 Euston Road, London, NW1 2DA UK; 2 Clinical and Research Informatics Unit, National Institute for Health and Care Research University College London Hospitals Biomedical Research Centre, University College London Hospitals NHS Foundation Trust, London, UK; 3 Centre for Medicines Optimisation Research & Education, University College London Hospitals NHS Foundation Trust, London, UK; 4 Research Department of Practice and Policy, University College London School of Pharmacy, London, UK; 5 Centre for Medicines Optimisation Research and Education, University College London Hospitals NHS Foundation Trust, London, UK; 6 University College London Interaction Centre (UCLIC), University College London, London, UK; 7 National Institute for Health and Care Research Biomedical Research Centre, South London and Maudsley NHS Foundation Trust, London, UK; 8 Department of Biostatistics and Health Informatics, Institute of Psychiatry, Psychology and Neuroscience, King’s College London, London, UK; 9 Postgraduate Institute of Medicine, Colombo, Sri Lanka; 10 Department of Cardiology, Amsterdam University Medical Center, Amsterdam Cardiovascular Sciences, University of Amsterdam, Meibergdreef 9, Amsterdam, AZ 1105 The Netherlands; 11 Cambridge University Hospitals NHS Foundation Trust, Cambridge, UK

**Keywords:** point of care, natural language processing, Diagnoses, Terminology, SNOMED CT

## Abstract

**Background:**

Well-organised electronic health records (EHR) are essential for high quality patient care, but EHR user interfaces can be cumbersome for entry of structured information, resulting in the majority of information being in free text rather than a structured form. This makes it difficult to retrieve information for clinical purposes and limits the research potential of the data. Natural language processing (NLP) at the point of care has been suggested as a way of improving data quality and completeness, but there is little evidence as to its effectiveness. We sought to generate such evidence by developing an open source, modular, configurable NLP system called MiADE, which is designed to integrate with an EHR. This paper describes the design of MiADE and the deployment at University College London Hospitals (UCLH), and is intended to benefit those who may wish to develop or implement a similar system elsewhere.

**Results:**

The MiADE system includes components to extract diagnoses, medications and allergies from a clinical note, and communicate with an EHR system in real time using Health Level 7 Clinical Document Architecture (HL7 CDA) messaging. This enables NLP results to be displayed to a clinician for verification before saving them to the patient’s record. MiADE utilises the MedCAT library (part of the Cogstack family of NLP tools) for named entity recognition (NER) and linking to SNOMED CT, as well as context detection. MedCAT models underwent unsupervised and supervised training on patient notes from UCLH, achieving precision of 83.2% (95% CI 77.0, 88.1), and recall of 85.2% (95% CI 79.1, 89.8) for detection of diagnosis concepts. In simulation testing we found that MiADE reduced the time taken for clinicians to enter structured problem lists by 89%. We have commenced a trial implementation of MiADE at UCLH in live clinical use, integrated with the Epic EHR at UCLH.

**Conclusions:**

We have developed an open source point of care NLP system and successfully integrated it with the EHR in live clinical use at a major hospital. Simulation testing has shown that our system significantly reduces the time taken for clinicians to enter structured diagnosis codes.

**Supplementary Information:**

The online version contains supplementary material available at 10.1186/s12911-025-03195-1.

## Background

### Benefits of structured information in electronic health records

Structured data in electronic health records (EHR) plays a crucial role in enhancing patient safety and facilitating decision-making processes. It enables the provision of important information, such as alerts for medication allergies and interactions, as well as reminders for monitoring chronic conditions [1]. Structured information is also vital for research and secondary data uses [2]. A randomised controlled trial has shown that structured problem lists lead to better and faster clinical decision-making [3].

However, much of the information in today’s records is in free text rather than in a structured form [4, 5], and is difficult to use clinically or for research. For example, an audit in UCLH during the COVID-19 pandemic found that only 62% of diagnoses for inpatients were included in problem lists [4]. Structured recording of diagnoses and other key items of clinical information is recommended by national guidance from the Professional Record Standards Body (PRSB) [6]. Widespread use of controlled clinical terminologies such as SNOMED CT (Systematized Nomenclature of Medicine Clinical Terms) [7] enables clinical concepts to be recorded in a consistent way, but only if clinicians are able to use the system easily. It can be onerous and time-consuming for clinicians to enter detailed structured information in many EHR systems [5], and time spent on data entry can affect the human experience of clinical consultations [8, 9].

### Role of natural language processing at the point of care

Natural language processing (NLP) can be used to convert unstructured text into structured data [10], but the semantic nuances of human language mean that this process is prone to error, and requires clinician validation before the information can safely be used for clinical decision making.

However, there is sparse published research on NLP at the point of care, in contrast to the extensive literature on retrospective NLP of healthcare text [10–12]. Havrilla et al. implemented a point of care application for entry of Human Phenotype Ontology (HPO) terms in a paediatric academic medical centre, which reduced data entry time from 15 to 5 minutes per patient [13]. Brigham and Women’s Hospital developed an NLP system for allergies integrated with Epic NoteReader [14]. There are commercial systems for point of care SNOMED CT coding marketed by Solventum [15], Nuance [16] and others, but limited published data of their effectiveness. It is also unknown how such systems should be best configured to prioritise the most important items of structured information, and avoid the clutter of irrelevant or unimportant results.

**Fig. 1:**
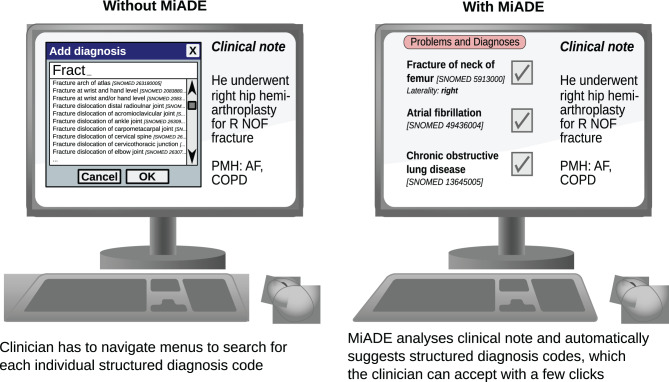
Electronic health record user interface, showing how MiADE (medical information AI data extractor) can assist structured data entry

Our study aimed to address this evidence gap by developing, implementing and evaluating a novel point of care NLP system called MiADE (Medical information AI Data Extractor). MiADE provides an alternative way for clinicians to enter SNOMED CT diagnosis codes by automatically generating suggestions for the clinician to validate, rather than having to browse the SNOMED CT dictionary to find the appropriate concepts (see Fig. 1). The hypothesis is that MiADE will improve the completeness of structured data by making it easier and quicker for clinicians to enter the data. This will hopefully improve clinical decision making and patient safety, and also benefit downstream uses of patient data such as research.

## Implementation

We developed a modular, open source NLP system called MiADE which is able to communicate with an EHR and process clinical notes in real time, returning suggestions to the EHR for display to the clinician. The clinician can use the EHR user interface to accept or reject the MiADE suggestions and reconcile them with existing information.

MiADE uses the open source MedCAT program [17], part of the Cogstack family of NLP tools [18], as its named entity recognition (NER) tool. MiADE does not have its own user interface but communicates with an EHR using data standards Health Level 7 Clinical Document Architecture (HL7 CDA) messaging, and can be readily adapted to use Fast Healthcare Interoperable Resources (FHIR). At UCLH, the user interface consisted of the ‘NoteReader’ component of the Epic EHR.

**Fig. 2:**
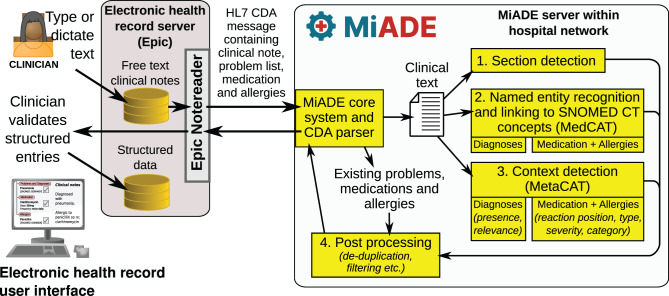
Schematic showing the overall architecture of the MiADE system

The system architecture is shown in Fig. 2, which illustrates how the key functions of the system link together:

1. Section detection
2. Named entity recognition and linking to SNOMED CT concepts (MedCAT)
3. Context detection (MetaCAT)
4. Post-processing

### Section detection

While the prose of clinical documents is hugely variable, we found that clinicians often write diagnoses, problems or medications in list-like formats within sections with a meaningful header. The context of information within such sections is much easier to process than in prose. We therefore designed a text cleaning and paragraph detection process which can detect section headings based on a configurable set of regular expressions (regex). Annotated outputs are then post-processed according to the paragraph type, and only relevant concepts are returned.

### Named entity recognition and linking to SNOMED CT concepts (MedCAT)

MiADE uses the open source MedCAT [17] library as its core NER algorithm. MedCAT is a self-supervised machine learning algorithm for extracting concepts using any concept vocabulary, available as an open source python package. MedCAT demonstrated superior performance on extraction of Unified Medical Language System (UMLS) concepts compared to SemEHR [19], Bio-YODIE [20], cTAKES [21] and MetaMap [22]. MedCAT models are trained unsupervised on a large text corpus to learn the context surrounding concept mentions in order to perform disambiguation. Supplementary supervised training can be carried out to help MedCAT learn additional synonyms and positive and negative examples of context. We have an active collaboration with MedCAT developers, and UCLH has already deployed MedCAT for retrospective clinical research [18].

#### MedCAT concept database and training

We built and trained separate models with different concept databases (CDB) for problems and medications / allergies, to allow each algorithm to be developed and implemented independently. We used a set of R scripts and the “Rdiagnosislist” R package to process SNOMED CT dictionaries and prepare the concept databases [23], using the UK SNOMED CT May 2022 version. The scripts and data are available in the miade-datasets GitHub repository (https://github.com/uclh-criu/miade-datasets/).

The problems concept database included symptoms, diagnoses, and important aspects of social history (e.g. care needs and housing), but not family history concepts, allergy concepts, normal findings or high-level concepts that are not relevant for a problem list, such as ‘Disease related state’. Acronyms expressed in SNOMED CT concept descriptions and a small number of common synonyms were added from a manually curated list.

The medication and allergy concept database included substances, symptoms that could be manifestations of allergic reactions, and medications from the Dictionary of Medicines and Devices (dm+d) [24] that are Virtual Therapeutic Moieties (VTMs) or Virtual Medicinal Products (VMPs).

MedCAT models are trained in unsupervised and supervised steps [17]. The unsupervised training step takes in a corpus of free-text documents and the CDB and learns the vector embedding representation of the context of each concept. This context representation is used to differentiate concepts when an ambiguous term is detected. For this step, we used a corpus of 800,000 UCLH clinical notes (mostly progress notes and discharge summaries) from April 2019 onwards (which is when the Epic EHR was installed at UCLH).

The supervised training process involves manual annotation to “teach” MedCAT to avoid incorrect annotations (i.e. words that do not have the same meaning as the concept they are linked to), and to learn additional correct synonyms and context. MedCAT can be trained to improve its performance in a particular domain of interest.

We chose discharge summaries as the initial focus for this project because of the clinical priority to improve handover between hospital and primary care, and to improve clinical coding and reimbursement. We therefore extracted a random subset of 400 discharge summaries for training from the UCLH corpus described earlier. These documents were annotated with SNOMED CT concepts by three clinically trained health informaticians (R.W., J.W., M.M.N.S.D.), using the MedCATtrainer tool provided by Cogstack [25], following a set of annotation guidelines (See Supplementary Text). We extracted a separate random subset of 50 discharge summaries for testing, which underwent double independent annotation. In cases of disagreement, annotations were reconciled manually by a third annotator.

### Context detection (MetaCAT)

MedCAT includes the ability to train small bidirectional long short-term memory (Bi-LSTM) neural network models to detect the context of concepts that are recognised. These are known as MetaCAT models, and can be trained using the same annotated data as the supervised training step of the MedCAT model. MetaCAT models are trained with standard neural network training procedures and evaluated with precision, recall, and F1 scores.

We trained MetaCAT models for the **Presence**, **Relevance** and **Laterality** of problems. For medications and allergies, we trained models to classify the position of a reaction mention relative to the substance **ReactionPos**, whether a substance is an allergen or a medication that is being taken **SubstanceCategory**, whether an adverse reaction risk is an allergy or intolerance **AllergyType**, and **Severity** of allergies (see Table 1). We carried out the training with the concept of interest (problem, medication, substance or reaction) replaced by a generic placeholder token, to prevent the models from incorrectly inferring context from the concept itself (e.g. to avoid assuming a concept is suspected because it frequently appears suspected in the training data).

**Table 1:** Meta-annotations for different models

Meta-annotation	Model	Description	Classes
Presence	Problems	Indicates the presence of a concept	confirmed, negated, suspected
Relevance	Problems	Indicates relevance of a concept	present, historic, irrelevant
Laterality	Problems	Indicates laterality of a concept; none if no laterality exists	left, right, bilateral, none
ReactionPos	Meds allergies	Indicates where the position of the concept relative to its nearest substance; none if the concept is not a reaction or no related substance	before_substance, after_substance, none
SubstanceCategory	Meds allergies	Indicates what type of substance the concept is; not_substance if the concept is not a substance	taking, adverse_reaction, not_substance, irrelevant
AllergyType	Meds allergies	Indicates what type of allergy the substance concept is; unspecified if no mention	allergy, intolerance, unspecified
Severity	Meds allergies	Indicates the severity of the allergic reaction to the substance concept; unspecified if no mention	mild, moderate, severe, unspecified

Outputs from the MetaCAT model are extracted into a MetaAnnotation class and returned as an attribute to the main concept. MiADE then applies a set of logic rules to the meta-annotations in combination with the section type to decide the final context of a concept. For example, if a concept is detected as “irrelevant” in a “previous medical history” paragraph, the meta-annotation is converted to “historic”, as the section heading takes precedence over the meta-annotation. Text without section headings is detected as “prose” paragraphs, and MiADE can either return or ignore concepts detected in prose paragraphs depending on the configuration setting structured_list_limit. This parameter limits the number of concepts that may be returned from prose. In the UCLH implementation it was set to −1 to ignore all concepts in prose, based on clinician feedback that it was preferable to avoid returning too many irrelevant concepts.

#### Synthetically augmented data for training MetaCAT models

One challenge we encountered in training the MetaCAT models was imbalanced classes in the annotated dataset: annotations of “suspected”, “historic”, and “irrelevant” occurred much less frequently than those of “confirmed” and “present”. To address this, we created additional synthetic training data using specially curated patterns of common phrases for expressing negation, suspected conditions, historic conditions, and allergies to augment the annotated dataset.

To facilitate the transparency and reproducibility of the training process with the synthetically augmented dataset, a *streamlit* dashboard was created to manage the dataset and model training process. This provides the ability to interactively review and modify the quantity of annotated data and synthetic data required in the training dataset. It can also automatically balance the class weights, version training parameters, and quickly visualise confusion matrices against the test set. The dashboard has been incorporated in the MiADE package.

### Post-processing

The core MiADE pipeline contains the NoteProcessor, Annotator, and DosageExtractor modules. The pipeline takes in a Note object and returns a list of Concepts. Functionalities to clean text and detect paragraph section headings are provided within the Note object. NoteProcessor acts as the main Application Programming Interface (API), to which the implementer can add MedCAT models to using the .add_annotator() method. The Annotator class wraps a single MedCAT model and processes the outputs from the model using custom post-processing algorithms we have developed. For usage with medication concepts, a DosageExtractor module is also provided, which extracts medication dosages and converts the output to CDA-compatible format. MiADE performs de-duplication of all concepts returned.

The MiADE processing pipeline is open source and available for use with different MedCAT models. To initialise MiADE, the implementer needs to provide a directory of MedCAT models, trained to extract concepts of different types (problems, medications, and allergies) and fine-tuned on local text datasets. Post-processing components are configurable via a set of lookup dictionaries for the conversion steps described below, as well as the regex that are used to extract section headings. A filter list can also be specified to blacklist irrelevant concepts, which can be amended without having to retrain the MedCAT model. The concept database and lookups used in our MiADE study are available on the public ‘miade-datasets’ GitHub repository [26].

Depending on need, MiADE pipeline components can be disabled in the configuration file e.g. for a use case that does not require certain post-processing steps such as dosage extraction or medication conversions. Implementers are therefore able to create plug-and-play processing pipelines with MedCAT models.

#### Post-processing of problems

Concepts detected with “negated”, “suspected”, and “historic” contexts are converted to corresponding SNOMED CT terms via lookup dictionaries, e.g. *fever* with a “negated” context is converted to the concept *apyrexia*. Concepts without conversion matches and irrelevant concepts are filtered out. Our implementation converts historic procedures to “history of” concepts (e.g. ‘History of coronary artery bypass graft’) but leaves historic diagnoses unchanged. This behaviour can be easily configured by custom lookup tables.

#### Post-processing of medications and allergies

The default MiADE algorithm extracts medications and allergies from a single MedCAT model, then differentiates and assigns medication and allergy type labels based on meta-annotation results.

**Fig. 3:**
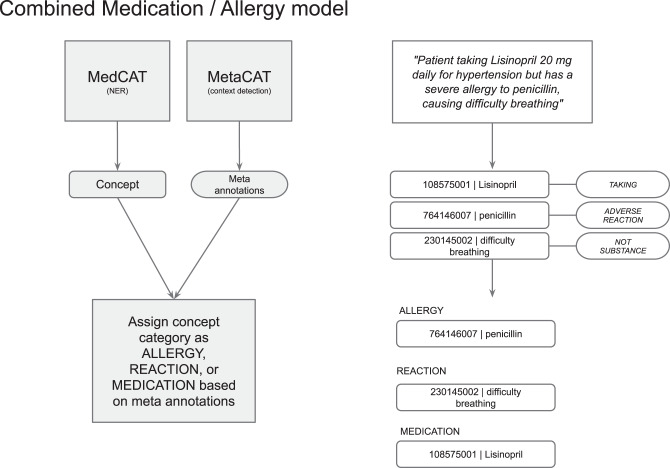
NER of medication and allergy model with example workflow

First, the detected concept is processed as either a medication (“taking”), allergy (“adverse reaction”) or not a substance (“not substance”, such as a reaction) based on the meta-annotations. The concept is then validated and converted to a SNOMED CT subset in each category if appropriate (see Fig. 3). This can be configured based on different implementation and use cases.

**Fig. 4:**
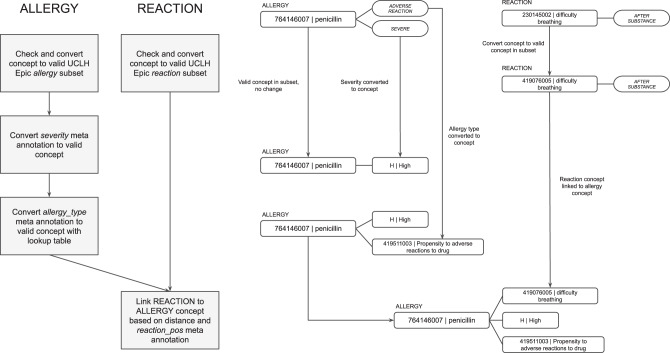
Post-processing of allergy concepts with example workflow

MiADE then performs linkage of the allergen to the allergy type, severity, and reaction. A single allergy entry is returned as an allergen SNOMED CT concept linked to concepts for allergy type, severity, and reaction in the CDA sent to the EHR (see Fig. 4). For each allergen substance detected, the severity is extracted through MetaCAT models and converted to appropriate SNOMED CT codes. The type of adverse reaction risk is also extracted through the MetaCAT model as either “intolerance”, “allergy”, or “unspecified”. An overall SNOMED CT concept for the allergy record type (e.g. “Food intolerance”) is chosen based on the adverse reaction risk type and substance type. Lastly, the reaction is detected by the core MedCAT model and linked to the closest allergen concept, based on whether the meta-annotation for the reaction concept is “before substance” or “after substance”.

**Fig. 5:**
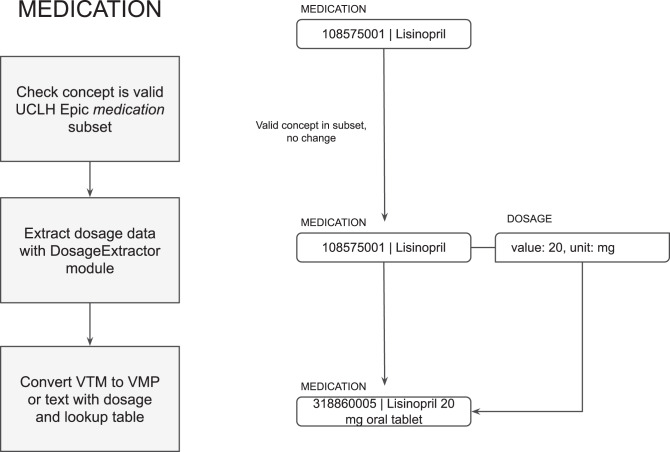
Post-processing of medication concepts with example workflow

Medications are detected either as Virtual Medicinal Products (VMP) if the dose form is stated, or Virtual Therapeutic Moieties (VTM) if only the substance is stated. The dosage is detected using the DosageExtractor algorithm described in the next section. VTMs may not be valid for return to the EHR depending how the EHR drug dictionary is set up, so MiADE includes a conversion table to convert them to VMPs for common doses of oral tablet medications. If the conversion is not possible, they are returned as text, in which case the clinician will need to select an appropriate VMP manually when reconciling the NLP output. Dosages are returned as CDA-compatible dosage information, where available (see Fig. 5).

#### Extraction of medication dosages

**Fig. 6:**
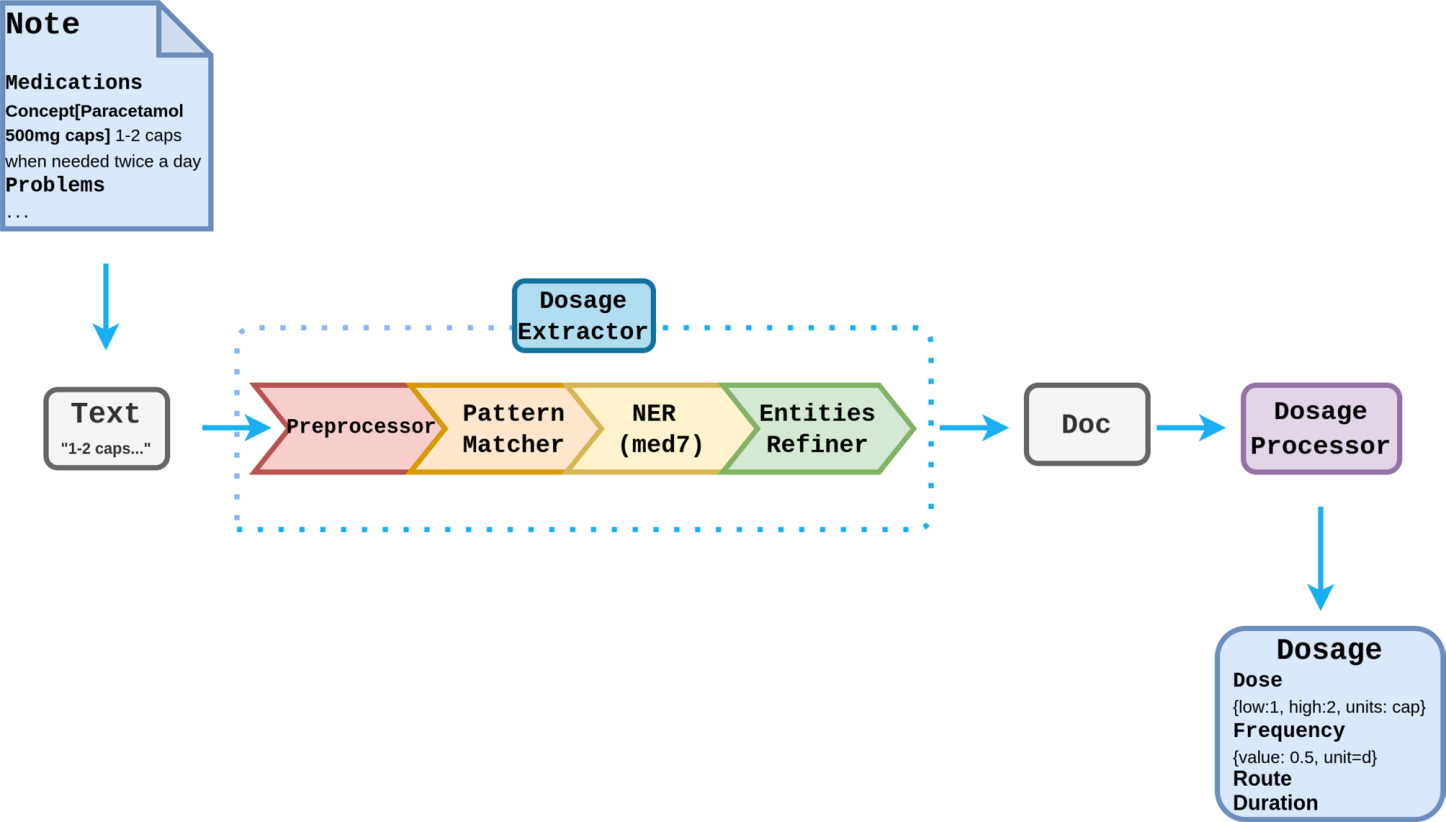
Pipeline design for drug dosage extraction

The first stage in dosage extraction is to identify words and phrases that constitute components of the dose. This is carried out using the med7 NER model, a *spaCy* neural network-based named entity recognition system for prescription components, trained on MIMIC III [27]. A rule-based algorithm was developed based on the CALIBERdrugdose R package [28] to calculate structured medication dosages. The dosage extractor is built as a *spaCy* pipeline and can be configured through the main MiADE API or used independently (See Fig. 6).

##### Pre-processing

The pre-processing steps are adapted from CALIBERdrugdose algorithm [28], which involves using two lookup dictionaries (*singlewords* and *multiwords*) to standardise words and expressions in the text and correct common spelling mistakes. This is followed by a *NumbersReplace* step to standardise the way quantities are expressed in the text (e.g. “2 ×200 mg” becomes “400 mg”, “1/2” becomes “0.5”).

##### Pattern matching

The Pattern Matcher step matches structural sentence patterns and performs rule-based entity tagging to disambiguate dosage information within the text, particularly dosage instructions that follow a specific format e.g. “*1 tablet to be taken twice a day, 30 tablets*”. It then uses the patterns lookup table from CALIBERdrugdose to extract dosage results and stores the results as an attribute in the *spaCy* Doc object.

##### Named entity recognition

The Med7 model is used to extract the labels: Dosage, Drug, Duration, Form, Frequency, Route, Strength. As not all labels extracted by Med7 are needed, some rule-based refinements are made to the NER results:

- Consecutive labels with the same tags are combined
- Drug and Strength labels are ignored (detected as part of VTM / VMP concepts by the main algorithm)
- Strength labels are combined with Dose labels if they are consecutive
- Drug labels are relabelled Form if they follow Dose (drug should be in concept name and should not appear in dose string)

##### Composition of a CDA dosage class

The processor takes the lookup and NER results from DosageExtractor and parses them into the CDA-compatible Dosage class:

- Dose [quantity, unit, low, high]
- Frequency [value, unit, low, high, standard_deviation, institution_specified, precondition_asrequired]
- Duration [value, unit, low, high]
- Route [code, displayName]

### Testing

We tested the following aspects of MiADE before deploying it in UCLH. First, we tested the accuracy of the MedCAT NLP models for detecting SNOMED CT diagnosis concepts, to ensure that the model training was adequate. Second, we carried out user acceptance testing of the complete pipeline integrated with EHR to ensure that it was usable by clinicians and behaving as expected. Third, to verify that the system can potentially save time for clinicians, we compared the time taken to enter a problem list with and without MiADE for simulated patients.

It was not possible to deploy medications and allergies initially because of issues with the EHR integration, hence model testing has not yet been performed for this functionality. Additional work is required to validate the medication and allergy pipeline before it is ready for clinical use.

#### Performance of the problems MedCAT model

We assessed the performance of concept detection for problems (symptoms and diagnoses) by testing the MiADE MedCAT model on a gold standard test set of 50 randomly selected discharge summary notes from UCLH, across a range of clinical specialties. These notes were double annotated by health informaticians and then manually reviewed to resolve any discrepancies.

We evaluated the MiADE problems algorithm on three components: concept detection, context detection, and full pipeline performance. The concept detection refers to the NER performance of MiADE, assessing the number of concepts detected irrespective of the metadata returned from the context detection algorithms. Context detection for problems ascertains affirmation status (affirmed, negated or suspected), temporality and relevance. The full pipeline performance assesses whether MiADE correctly suggests a concept given the full pipeline of concept detection and applying post-processing and filtering logic based on the metadata from context detection. For each component we quantified the performance with precision, recall, and F-1 score. For all evaluations we disabled the paragraph detection and deduplication components to assess the performance of the MedCAT / MiADE algorithm on interpreting prose. Each component was evaluated on two filtered subsets of concepts - ‘Symptoms + Diagnoses’ (concepts with the SNOMED CT semantic types “Finding” or “Disorder”, omitting administrative concepts), and ‘Diagnoses Only’ (mostly SNOMED CT “Disorder” concepts, with a few clinically important findings such as “Seizure” included).

The concept detection and full pipeline performance were evaluated on a per-document basis, without positional matching of concepts. A SNOMED CT concept that was detected by MiADE but not present in the gold standard is classed as a false positive, and vice versa for false negative.

To evaluate context detection, we needed to match up concepts in the MiADE output with those in the annotated gold standard. We were unable to do this directly because of shifts in the concept spans from the text cleaning and pre-processing stages. Instead, for each document we first cross-joined the set of concepts returned by MiADE with the gold standard set of concepts. We then used a custom fuzzy match algorithm based on a combination of positional matching and string similarity to identify spans that corresponded to the same concept. From this set we checked if the SNOMED IDs of each matched concept matched, giving the final set of correctly detected and suggested concepts. The context detection evaluation did not include concepts that were identified as false positives, false negatives, or were not matched by the fuzzy-matching algorithm.

#### User acceptance testing

MiADE was installed on a server linked to a test installation of Epic. Clinician investigators working on MiADE tried out the system with simulated patient histories to ensure that the user interface worked as expected. This was essential because the manner in which Epic renders CDA data is frequently lacking in comprehensive documentation, necessitating a process of trial and error to verify that the information is displayed as intended.

#### Testing speed of data entry

We set up a data entry task for clinicians and timed them using the default EHR method of entering SNOMED CT concepts (using the EHRs terminology browser) and using MiADE (where the problems would be auto-suggested for clinician verification; see Fig. 1). Clinicians were asked to perform the task on a set of 20 simulated clinical notes each containing a problem list with 10 problems. The problem names were SNOMED CT synonyms for diagnosis concepts randomly selected from the frequency distribution of SNOMED CT concepts in UCLH problem lists in 2019–2021. This task was designed to ascertain the maximal possible time saving using MiADE, as the terms were SNOMED CT names without variations or mis-spellings and would be accurately detected by MiADE.

Study participants were five clinicians who had experience of using Epic, with a range of seniority and familiarity with digital technology.

### Integration with EHR

We deployed and integrated MiADE into the Epic EHR system at UCLH. In addition to the core open source MiADE library, we also developed a server and a CDA parser for integration into EHRs. The MiADE server is deployed as a Docker container on hospital internal network and communicates with Epic over secure HTTPS. It implements the Simple Object Access Protocol (SOAP) method ProcessDocument, which is invoked by Epic to send over a CDA document that contains the note entered by the user in Epic NoteReader. To trigger this request, the user selects or toggles the “Send to NoteReader” button when they are in the Epic NoteReader interface. The input CDA is parsed and processed by the MiADE server, and a response CDA document containing the extracted data is returned to Epic.

We implemented the SOAP endpoint invoked by Epic as a Web Server Gateway Interface (WSGI) sub-application mounted to a *FastAPI* app at /notereader/, so that the server is able to receive both RESTful and SOAP requests. This modularisation lends extensibility to the server implementation and allows possibility of integration with different EHR systems, including the option to receive and return data in FHIR format.

### Pathway to deployment

MiADE was initially incorporated into a test environment of Epic at UCLH, which is not linked to the live clinical system and does not include actual patient data. Prior to production deployment of MiADE, we load tested our servers using the load testing framework *locust* for a maximum capacity of 100 concurrent users to ensure the server is able to support the load of the feasibility trial. The server was distributed across 8 worker nodes (one for each CPU core), with load balancing handled by *nginx*. Test request data used were CDA document requests with varying lengths of structured data and progress note lengths.

We developed a clinical safety case following the NHS digital safety standards [29, 30]. We performed a risk evaluation by convening hazard workshops with clinicians, health informaticians and software engineers. A hazard log was compiled based on the workshop discussions. Risks fell into three broad themes: risks due to failure of the MiADE software (crashes/data leaks etc.), risks due to the effect of the integration of MiADE on the EHRs (uncovering bugs in an otherwise unused function of the EHRs), and clinician behaviour change (failure to properly vet suggestions from MiADE).

We worked with the EHR specialist team at the hospital to carry out a series of tests to validate that the communication between the EHRs and MiADE was working, data was correctly placed into the medical record, and that the interface functioned as expected.

Following testing and hazard workshops, a safety case was submitted to the Trust Clinical Safety Committee, and subsequent Trust approvals were sought for live deployment within the context of an evaluation study for up to 100 users. The initial live implementation was limited to diagnosis only due to unresolved issues with the Epic EHR integration for medications and allergies.

## Results

### Performance of the MiADE MedCAT model for problems

Performance of the MiADE MedCAT model for diagnoses was tested on a double-annotated gold standard dataset of 50 discharge summaries containing 551 symptom or diagnosis concepts.

#### Concept detection - NER

Concept detection refers to pure NER performance by the MedCAT models used in MiADE, with additional filtering for subsets only. Precision was 78.9% (95% Confidence Interval (CI) 74.5, 82.7) and recall was 85.0% (95% CI 80.9, 88.4), giving an F-1 score of 0.82 for ‘Symptoms + Diagnoses’. The number of false positive suggestions was reduced in the ‘Diagnoses Only’ subset with the more restrictive filtering, giving an improved precision of 83.2% (95% CI 77.0, 88.1), a recall of 85.2% (95% CI 79.1, 89.8), and an F-1 score of 0.84; see Table 2. Inter-annotator agreement for the correctness of the chosen SNOMED CT concept (Cohen’s kappa) was 0.704.

**Table 2:** Performance of named entity recognition (NER) and full pipeline (NER with context detection) for SNOMED CT symptoms and diagnoses (clinical findings) and diagnoses (disorders) only

Evaluation task	True positive	False positive	False negative	Precision % (95% CI)	Recall % (95% CI)	F1
NER (Symptoms + Diagnoses)	295	79	52	78.9 (74.5, 82.7)	85.0 (80.9, 88.4)	0.82
NER (Diagnoses Only)	144	29	25	83.2 (77.0, 88.1)	85.2 (79.1, 89.8)	0.84
Full pipeline (Symptoms + Diagnoses)	156	101	54	60.7 (54.6, 66.5)	74.3 (68, 79.7)	0.67
Full pipeline (Diagnoses Only)	79	43	26	64.8 (55.9, 72.7)	75.2 (66.2, 82.5)	0.70

Manual inspection of the errors revealed that the majority of false positives were due to misinterpretation of acronyms or words with multiple meanings, such as “analgesia” (which MedCAT interpreted as a finding of “No sensitivity to pain” but was intended to mean “pain medication”). The majority of false negatives were due to mis-spellings or variants of phrasing that did not correspond to SNOMED CT synonyms.

#### Context detection - MetaCAT models

The context detection refers to the combined performance of the MetaCAT models within MiADE and the subsequent post-processing pipeline of filtering and conversion based on the context annotations from MetaCAT. A detected concept is suggested if:

$$\eqalign{& {\rm{Presence}} = \cr & {\rm{Confirmed}} \wedge \cr & ({\rm{Relevance}} = {\rm{Present}} \vee {\rm{Relevance}} = {\rm{Historic}}) \cr} $$

Where ‘Presence’ and ‘Relevance’ refer to annotation outputs from the respective trained MetaCAT models. This was calculated only on the 261 concepts (out of 551) that had been correctly detected in the gold standard dataset and where we were able to match up the concept in the MiADE output with the gold standard using our fuzzy-matching algorithm.

For the ‘Symptoms + Diagnoses’ subset, a total of 228 correctly identified concepts by MiADE were matched to the gold standard dataset, giving a precision of 82.5% (95% CI 76.7, 86.9), a recall of 98.9% (95% CI 95.9, 99.7), and an F-1 score of 0.9. For the ‘Diagnoses Only’ subset, a total of 121 concepts were matched to the gold standard, giving a precision of 80.7% (95% CI 72.7, 86.8). As there were no false negatives from the more restrictive filtering, the recall was 100% (95% CI 96.2, 100) and the F-1 score was 0.89 (Table 3).

**Table 3:** Performance metrics of MetaCAT models

Evaluation task	True positive	False positive	False negative	True negative	Precision % (95% CI)	Recall % (95% CI)	F1
Context detection (Symptoms + Diagnoses)	173	37	2	16	82.5 (76.7, 86.9)	98.9 (95.9, 99.7)	0.90
Context detection (Diagnoses Only)	96	23	0	2	80.7 (72.7, 86.8)	100 (96.2, 100)	0.89

#### Full pipeline

The full pipeline performance of MiADE includes the concept detection (NER), context detection (MetaCAT models), post-processing pipeline of filtering and conversion based on the context detection, and final filtering to ‘Symptoms + Diagnoses’ and ‘Diagnoses Only’ subsets of concepts.

The precision of the ‘Symptoms + Diagnoses’ subset was 60.7% (95% CI 54.5, 66.5) and the recall was 74.3% (95% CI 68, 79.7) with an F-1 score of 0.67. Like the NER results, the full pipeline ‘Diagnoses Only’ subset had fewer false positives, giving a precision of 64.8% (95% CI 55.9, 72.7), a recall of 75.2% (95% CI 66.2, 82.5) and an F-1 score of 0.7 (Table 2).

### Testing the speed of data entry

Five doctors - two Specialty Registrars, an Internal Medicine Training (IMT) doctor, a Foundation Year 2 (FY2) doctor and a Foundation Year 1 (FY1) doctor - participated in the speed test with simulated clinical notes. The results showed that MiADE and NoteReader reduced the time taken to enter problem lists by 89% compared to the default Epic interface (see Table 4).

**Table 4:** Comparison of time taken to enter 10 problems in the EHR using Epic’s default SNOMED CT browser versus MiADE with epic NoteReader

Participant	Time taken to enter 10 problems in Epic (seconds)	
	Default method	Using NoteReader with MiADE
1	146, 97	7, 12
2	106, 112	14, 13
3	135, 132	16, 11
4	108, 101	9, 10
5	160, 226	24, 28
Mean	132.3	14.4
Standard deviation	39.0	6.7

### Deployment

We successfully deployed MiADE integrated in a live EHR environment at UCLH. MiADE was deployed on a microservice architecture within UCLH internal network. This instance of MiADE runs on distributed CPU, but MiADE is also capable of running on GPU or scaled to a cloud-based approach using Kubernetes.

MiADE went live for internal investigators on the project on 14 February 2024 and became available to use for all trial users from 26 February 2024. From the period of 26 February 2024 to 8 May 2024, a total of 1645 documents were processed by MiADE, and 317 concepts were extracted and added to 111 patients’ records.

## Discussion

### Summary of main findings

We successfully developed an open source NLP system called MiADE and integrated it with the EHR at UCLH for an evaluation in live clinical use. MiADE is designed to integrate with EHRs to extract diagnoses, medication and allergies from a clinical note in real time and provide the results for immediate clinical verification. In simulation testing we found that MiADE can significantly reduce the time taken for clinicians to enter structured problem lists. We hope the information in this article will be useful for others proposing similar work in the future.

### Algorithm performance and clinical utility

We consider that the performance of the MiADE MedCAT model (83% precision, 85% recall for NER on diagnoses) is sufficient to be clinically useful, and these performance statistics are similar to those obtained using a range of methods on I2b2–2010 concept extraction benchmarks in a recent systematic review [31]. While some diagnoses may be incorrect and some may be missed, these are unlikely to cause safety issues because MiADE operates within a human-in-the-loop system; i.e. the EHR user interface requires manual review of MiADE suggestions (with an opportunity to correct them) before they are committed to the patient’s record.

The detection of context and relevance in prose was more difficult and reduced the F1 score to 0.67, so in production we configured MiADE to return only problems that are in a paragraph or section with a heading such as “Problem list” or “Diagnoses”. Clinicians can therefore use headings to lay out their clinical note for MiADE to work efficiently.

### Algorithm design

The core MiADE algorithm consists of MedCAT models, which perform NER + linking, and MetaCAT models, which augment concepts detected by the NER step with contextual information from LSTM-powered MetaCAT models. The MiADE system is modular, customisable, and portable to different hospital EHRs needs. It does this by allowing implementers to build plug-and-play pipelines of different MedCAT models and configure custom post-processing components.

The role of small models such as MedCAT has been questioned in the era of LLMs. Whilst LLMs have impressive reasoning ability, they are error-prone for medical coding tasks (e.g. they can hallucinate SNOMED CT concepts that do not exist) [32] and in general do not perform as well as smaller models on NER tasks [33]. They would require additional manual processing to ensure they return reliable results, in which case they would not have any significant advantage over smaller models. In addition, the resource and environmental cost of LLMs make them difficult and expensive to deploy, fine-tune, and scale. However, LLMs are superior at understanding context and can perform complex reasoning tasks [34, 35]. This could be used to improve the ability of MiADE to extract concepts from prose, and also to gather and generate more detailed structured information. Recent advances in LLM techniques such as Retrieval Augmented Generation (RAG) [36], chain-of-thought prompting [35] and agentic workflows [37] are promising approaches.

With this in mind, MiADE is designed to be modular and extensible, allowing components like the NER model or post-processing pipelines to be easily replaced with more sophisticated models or algorithms. The plug-and-play architecture of MiADE enables the creation of robust pipelines that combine fast, lightweight algorithms in simple components with LLMs for context-aware reasoning in more complex components. MiADE should serve as a foundation for building open, hybrid-approach data extractor pipelines that can be easily fine-tuned and integrated at different hospital EHRs. We believe this will be a significant advantage over existing monolithic enterprise solutions on the market, such as Solventum [15] and Nuance [16]. By leveraging the power of open source, our approach empowers the community to create customised versions of MiADE tailored to their individual needs. This flexibility and adaptability will set MiADE apart from the rigid, one-size-fits-all commercial offerings currently available.

### Challenges encountered

A particular challenge we encountered while developing MiADE for clinical deployment was working with local configurations of EHRs data. In the case of healthcare interoperability standards, function does not always follow form - although HL7 standards technically ensures interoperability between systems, we have found that local preferences in coding systems and configurations may deviate from the out-of-the-box template specified by the EHR provider’s developer resources for third-party integration, leading to inaccurate parsing of data. These variations in usage are often not well documented, and as a result, a disproportionate amount of time was needed to coordinate with different stakeholders and reverse engineer the behaviour of the reading and writing of data in the EHRs test instance. In other words, our experience indicates that the reality of existing EHR systems is that they are interoperable, but not *composable*. This may present more overhead for widespread roll-out and installation of MiADE at different sites, adding to the existing challenges of working with legacy systems.

Future implementers should also consider appropriate strategies for development infrastructure and Trusted Research Environments (TRE) optimised for the clinical deployment of machine learning and NLP workflows [38]. While accessing hospital systems via secure network is adequate for ad hoc development work and server hosting, the overhead and technical debt can quickly add up for work that involves data and model training, taking away from time that could be spent on setting up robust Continuous Integration / Continuous Delivery (CI / CD) software development and MLOps (Machine Learning Operations) best practices.

### Limitations

Although MiADE was developed with close consultation of clinicians and end-users, the user interface design and what can be presented to a user is limited by the fact that outputs have to adhere to the Epic NoteReader UI interface. For example, the MiADE backend has no control over the order in which the problems are displayed (unless explicitly coerced by adding numbering before the concept text), or the action buttons presented to users within the interface. To ensure user-friendliness and more wide-spread clinician adoption of the system, full control over user interface design should be considered. The interfacing method with Epic NoteReader also dictates that CDA must be used for communication between Epic and NLP vendors, which can be more limiting in terms of the flexibility of information communication than FHIR.

While the lightweight MedCAT NER models excel at quickly detecting well-written concepts, they have limited ability to understand the context of concepts, and can have difficulty with ambiguous text. The presence of unusual spellings and abbreviations in clinical text required us to augment the concept database manually, making the system less generalised. The text searching algorithm of the core NER models is vulnerable to bracketing mistakes, for example: “abnormalities found in lung, cancer not detected” might lead MedCAT to detect the concept “lung cancer”. The MetaCAT models that perform the context detection could similarly mistake nearby but semantically unrelated context when classifying a concept.

The MiADE post-processing algorithm, in particular medications and allergies, was written tightly-coupled to the peculiarities of how the local hospital instance stores medication concepts. As a result the post-processing algorithm may not be readily applicable to another location without adjustments to the code. We have designed the MiADE pipeline to be extensible to account for this, so that custom logic and data can be created and configured, together with MedCAT models trained on local text.

The MiADE testing reported in this paper has limitations. The NLP performance testing was based on a sample of a single type of clinical note (discharge summaries), and may not be representative of performance in specific clinical areas. The simulation speed test was artificial and may not represent real life performance because MiADE NLP may not work as well with real patient notes. However it was intended to demonstrate the need and theoretical maximal benefit of such a system.

### Recommendations for future work

This project takes an initial step towards making EHR user interfaces more intelligent and user-friendly in order to capture richer structured data at the point of care. There are further areas of development in the user interface design itself, such as enabling NLP suggestions to be displayed as the user types or dictates rather than on saving the document, as with NoteReader. Diagnoses are currently recorded as flat SNOMED CT concepts without attributes such as date, body, site, manifestations etc. which are included in openEHR [39] and FHIR [40] information models for diagnoses. More sophisticated NLP techniques such as LLMs can be used to detect complex concepts and their relationships, while maintaining accurate coding. There is also a need to understand how well models trained at one centre perform at another, and to what degree local training of models and tailoring of algorithms is required.

## Conclusions

We have successfully designed and implemented an open source NLP system to extract structured information from free text at the point of care, and integrated it with the EHR system in live clinical use at a major hospital. Simulation testing has shown that our system significantly reduces the time taken for clinicians to enter structured diagnosis codes, and it may therefore help to improve the completeness of clinical records without impeding clinicians’ workflow.

### Availability and requirements

The MiADE package version v1.0.7 used in this paper is available under the Elastic 2.0 licence. It can be accessed and used through the following means:

- **Project name**: MiADE
- **Project home page**: Detailed documentation is available at (https://uclh-criu.github.io/miade/).
- **Operating system**: Linux, macOS
- **Programming language**: Python
- **Licence**: Elastic 2.0
- **Any restrictions to use by non-academics**: Under the Elastic 2.0 licence, you may not provide the products to others as a managed service

## Electronic supplementary material

Below is the link to the electronic supplementary material.

Supplementary Material 1

## Data Availability

The patient data used for training NLP models may contain sensitive information and external access is therefore not possible. The trained NLP models may be shared with other NHS Trusts according to data sharing agreements. Datasets of SNOMED CT concepts, mappings and lookups used by the UCLH implementation of MiADE are available on GitHub (https://github.com/uclh-criu/miade-datasets/).

## Code availability

The MiADE package version v1.0.7 used in this paper is available under the Elastic 2.0 licence. It can be accessed and used through the following means:

• **PyPI (Python Packaging Index)**: MiADE is available as a Python package on PyPI (https://pypi.org/project/miade/). It can be installed using pip:

pip install miade= = 1.0.7

• **Source Code**: The latest version is available on GitHub (https://github.com/uclh-criu/miade).

**Documentation**: Detailed installation instructions, usage examples, and API reference, is available at: https://uclh-criu.github.io/miade/

• **Archive**: A static version of the code has been archived and assigned a DOI: [DOI 10.5281/zenodo.13323598]

• **Requirements**: MiADE is compatible with Python > = 3.8, < 3.12 and has been tested on Linux and macOS. A complete list of dependencies and version constraints can be found in the pyproject.toml and requirements.txt files in the GitHub repository.

• **Reproducibility**: The scripts and data used to train the MedCAT models and post-processing lookup data used in the MiADE implementation at UCLH is made available in the Github repository miade-datasets (https://github.com/uclh-criu/miade-datasets).

The “Rdiagnosislist” R package was used for processing of SNOMED CT dictionaries and preparation of concept databases for MiADE. The latest version is available on GitHub (https://github.com/anoopshah/Rdiagnosislist), licensed under GPL-3. The stable version is on the Comprehensive R Archive Network (https://cran.r-project.org/web/packages/Rdiagnosislist/index.html).

## Abbreviations

CDA: Clinical Document Architecture
CDB: Concept database
EHR: Electronic health record
FHIR: Fast Healthcare Interoperable Resources
HL7: Health Level 7
HPO: Human Phenotype Ontology
LLM: Large language model
LSTM: Long short term memory
MedCAT: Medical Concept Annotation Tool
MiADE: Medical information AI Data Extractor
NER: Named Entity Recognition
NHS: National Health Service
NIHR: National Institute for Health and Care Research
NLP: Natural language processing
SNOMED CT: Systematized Nomenclature of Medicine Clinical Terms
NLP: Natural language processing
UCLH: University College London Hospitals
UMLS: Unified Medical Language System
VMP: Virtual Medicinal Product
VTM: Virtual Therapeutic Moiety
