# Supplementary material for: Design and implementation of a natural language processing system at the point of care: MiADE (medical information AI data extractor)
Source: BMC Med Inform Decis Mak. 2025 Oct 7;25:365. doi: 10.1186/s12911-025-03195-1 (PMC12506320; doi:10.1186/s12911-025-03195-1)
Supplement: Supplementary file 1 — Supplementary Material 1 [file 12911_2025_3195_MOESM1_ESM.pdf]

NIHR | University College London Hospitals Biomedical Research Centre

NHS
University College London Hospitals
NHS Foundation Trust

MedCAT Trainer Annotation Guide for MiADE problems and diagnoses

User guide for MedCAT with Trainer interface as an NLP tool to annotate clinical notes for methods validation and improvement.

Authors: Leilei Zhu, Lukasz Roguski, Anoop Shah, James Brandreth

Last update date: 15/08/2024

For: Validators

This document describes how to use the MedCAT trainer tool to create manual annotations for training the problem / diagnosis algorithm for MiADE

A partnership between UCL and University College London Hospitals NHS Foundation Trust

UCL

MiADE GitHub: https://github.com/uclh-criu/miade

Introduction

What is MedCAT?
MedCAT stands for Medical Concept Annotation Tool (MedCAT). It is an open-source set of tools for unsupervised and supervised approach to Named Entity Recognition and Linking (NER+L), or in common terms NLP (natural language processing). NER means detecting the entity in the text (e.g. medical terms). While L means linking the recognized entity to a concept in a biomedical database (e.g. SNOMED CT).

What is MedCAT Trainer?
MedCAT Trainer is a user interface allowing to annotate the notes while refining the MedCAT NLP models. The main goal of the interface is to perform supervised training on the MedCAT NLP model assigned to the task.

MedCAT Annotation and Validation Guide

1. When opening a document, the key words are automatically highlighted.

Clinical Text in a nutshell:
The selected documents text, highlighted with each concept recognised by the configured MedCAT model. Highlighted spans of text indicate status of the annotation:

Grey: A User has not reviewed this span that has been recognised and linked by MedCAT to a CDB concept.
Blue: A User has reviewed the span and marked it as correct in terms of its linked MedCAT concept.
Red: A User has reviewed the span and marked it as incorrect in terms of its linked MedCAT concept.
Dark Red: A User has reviewed the span and marked it to terminate, meaning the text span should never again link to this text span.
Turqoise: A User has reviewed the span and marked it as an alternative linked concept. The user has used the 'Concept Picker' to choose the correct concept that should be linked.

2. Users are prompted with four choices.

- ***Correct***: correct SNOMED CT concept has been chosen
- ***Incorrect***: text does not relate to any relevant SNOMED CT finding / disorder concept
- ***Terminate***: stop showing the concept in the future (ignore)
- ***Alternative***: there is a better SNOMED CT concept match available

Diagnoses:

Patient Active Problem List
Diagnosis SNOMED CT(R)

- TIA (transient ischaemic attack) Transient cerebral ischaemia
- GERD (gastroesophageal reflux disease) Gastrooesophageal reflux disease
- Oesophagitis Oesophagitis
- Gastritis, erosive Erosive gastritis
- HH (hiatus hernia) Hiatal hernia
- Angioimmunoblastic lymphoma Angioimmunoblastic T-cell lymphoma

FBC:
Lab Test Results
Component Value Date

WHITECELL 6.30 09/01/2020

HAEMOGLOBIN 118 (L) 09/01/2020

HCT 0.346 (L) 09/01/2020

MCV 101.8 (H) 09/01/2020

PLATELET 179 09/01/2020

NEUTROPHILS 5.23 09/01/2020

Correct | Incorrect | Terminate | Alternative | Submit

Concept Summary

Annotated Text	TIA (transient ischaemic attack
Name	Transient ischemic attack (disorder)
Term ID	T-02100
Concept ID	S-266257000
Accuracy	1.00
Description	n/a

3. After selecting ***Correct***, or ***Alternative***, users are promoted to further qualify this concept under **Meta Annotation Tasks**. We are only interested in finding or disorder concepts that relate to the current patient.

Confirmed (default), Suspected, Negated

- Confirmed = confirmed or working diagnosis ('treat as')
- Suspected = possible, query, suspected, probable
- Negated = not, absent, no evidence of ...

Present (default), Historic, Irrelevant

- Present = default
- Historic = problems that would be considered 'past medical history', e.g. 'history of MI', 'previous stroke'
- Irrelevant = hypothetical or 'risk of', 'fear of', family history etc.

No laterality (default), Left, Right, Bilateral

- No laterality = laterality is irrelevant for this concept, or is not mentioned
- Left = e.g. left foot
- Right = e.g. right arm
- Bilateral = e.g. both kidneys

4. After selecting "***Alternative***"

If the user feels a more appropriate concept should be selected, after click ***Alternative***, a **Name** field will appear to allow user to select a more appropriate concept.

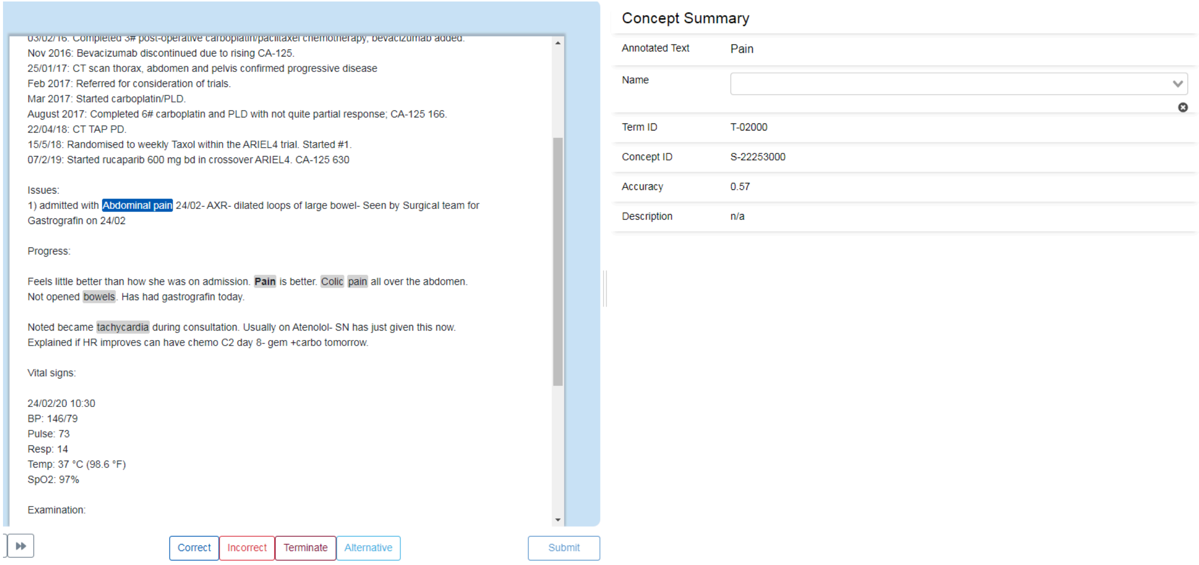

Users can perform the following:
Add Synonym: to add this annotation to the text span and link the selected concept. Enter the SNOMED CT concept preceded by 's-'
Cancel: (Shortcut esc): to cancel adding the annotation to the text.

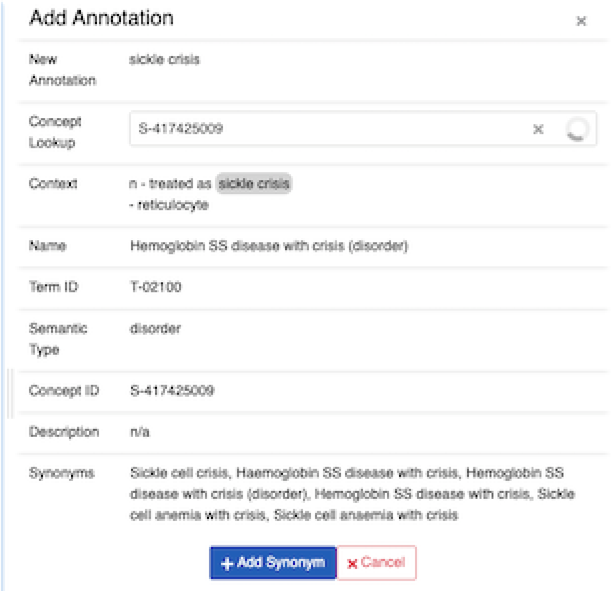

5. If a wrong concept was selected, you can correct them by doing this???

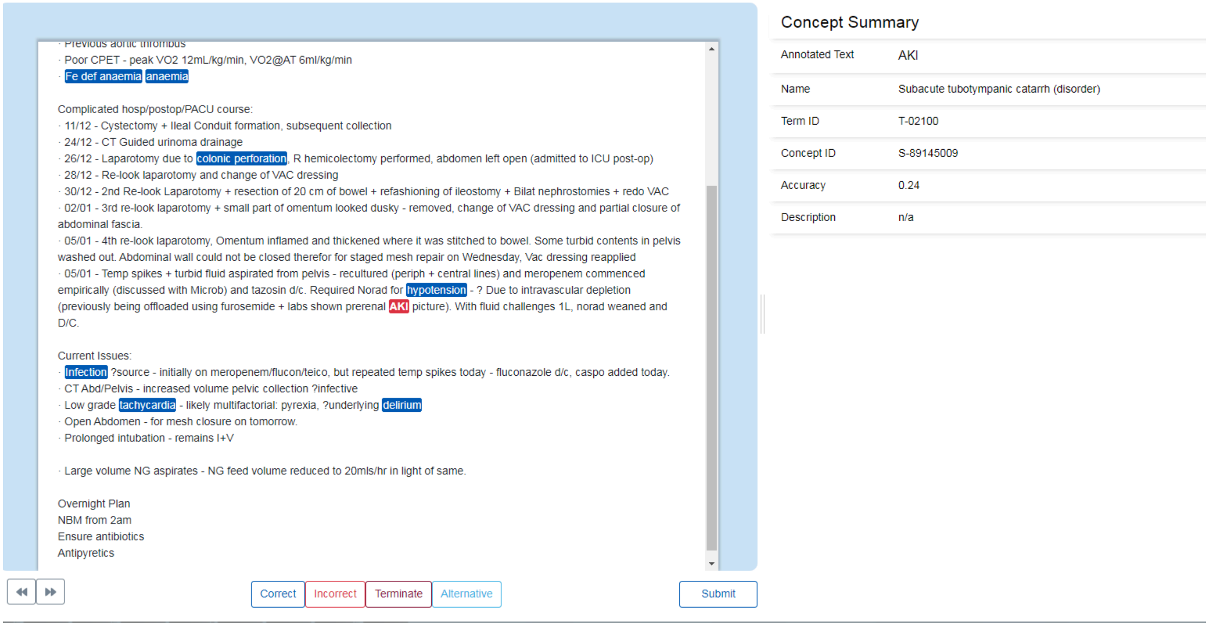

6. The "forward" and "backward" button on the left hand corner is to go to next and previous annotation.

7. When done with annotations, click **Submit** button to save the results.

8. In cases when there is nothing highlighted for annotation, just click **Submit** button to move forward to another document.

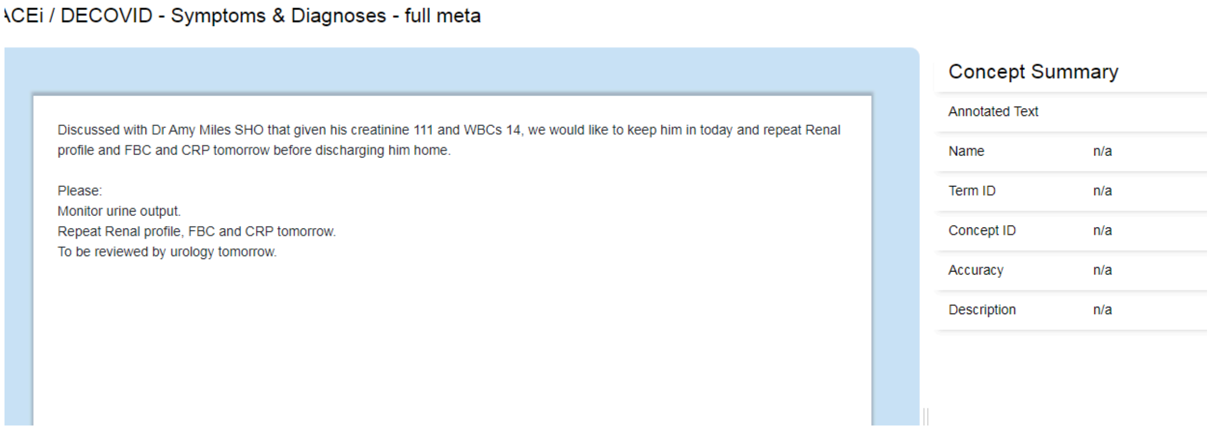

MedCAT Annotation and Validation protocol

Annotation protocol for calculating the accuracy of the algorithm

When performing annotation of clinical concepts in a project, the collected annotations provided by annotators need to be validated. Documents should be annotated by at least 2 annotators, as there would be cases where:

- Annotators disagree on a selected term for annotation (correct / incorrect) or an alternative term is proposed
- A new annotation was added by only one of the annotators as it was not detected by MedCAT NLP model
- Proposed meta-annotations attached to an annotated concept do not match

Hence, the annotation results need to be merged into a concise set of annotation with the annotations that are not agreed being resolved by an arbitrator.

The set of annotation with the disagreements resolved will be considered at the 'gold standard' and the NLP model will be refined using that set.

It is assumed that the arbitrator would be a person with a senior or comparable clinical background as the annotators to be able to resolve the dispute.

1/ Annotation protocol

1. A project answering a defined use-case is created with a selected set of documents.
2. In parallel annotators go through a set of documents and annotate the terms as defined in this document
3. At a certain stage (e.g. after annotating an agreed number of documents), the results are analysed, and validation is stared in parallel

2/ Validation protocol

1. Based on the previously proposed annotations a validation project is being created and with the same set of documents. The documents will only contain annotations for which there was disagreement.
2. The arbitrator will mark the concepts either as correct or incorrect. The same would apply for meta-annotations, selecting a valid one.

References

[1] MedCAT paper: https://arxiv.org/abs/1912.10166
[2] MedCAT Trainer paper: https://arxiv.org/pdf/1907.07322.pdf
[3] MedCAT Trainer article:
https://medium.com/data-science/medcattrainer-a-tool-for-inspecting-improving-and-customising-medcat-880a11297ebe

Updates:

- 21/04/2020 - Leilei: Initial version
- 22/04/2020 - Leilei: Extra questions
- 29/04/2020 - Lukasz: Refinement + added section about protocols
- 14/06/2022 - Anoop: Adapting for MiADE version of MedCAT
- 15/08/2024 - James: Cleaning for publication
